# Ventilation

**Published:** 1900-03-10

**Authors:** 


### Ventilation.

A good deal of discussion has lately taken place in regard to the question whether, in arranging for the ventilation of a living room or a hospital ward, air should be made to leave the apartment at the top or at the bottom, a matter which is of considerable importance in the planning of a building. The problem of ventilation in this climate is dominated by two facts—first, that whenever the weather is such as to prevent the free opening of windows the air within the room is warmer and lighter than the air outside, and therefore tends to float away upwards; and, second, that in our climate the outer air is practically always in motion, and that the effect of wind is to give rise to an up-draught in all chimneys and ventilating tubes across which it blows. Thus what one may call the natural tendency is for the air within the room to escape into the air outside in an upward direction, and it is clear that in any arrangement for making the warm air escape at the bottom of the room instead of at the top some compelling force must be employed to overcome this tendency, which is impressed upon it by physical laws. That being so it is for those who advocate placing the outlets near the floor to give some strong reason for a plan which involves such an expenditure of energy, and of that which in this world sets energy in motion, namely, money. In practice this compelling force is employed only in two systems of ventilation—first in that by open fires, in which the extravagance of heating by radiation is at least to some extent compensated for by the fact that by means of the draught up the chimney a very large amount of air is constantly removed, and that ventilation is ensured under all conditions so long as the fire is alight; and, secondly, in that method which has gained for itself the name of the "plenum system," in which air under a certain regulated pressure is driven throughout the building. According to this system the whole building is made as far as possible air-tight, with the exception of the appointed inlets and outlets. Air is drawn in at certain places, is warmed to the proper temperature, and is driven into the building at a certain pressure—"plenum"—by means of which it is forced to take a certain course to its appointed outlets.

This pressure together with what may be termed the syphonic action, which results from the closed construction, are sufficient to entirely overcome any reverse current set up by differences of temperature, and it is usual to place the outlets at the floor level. For this, however, there is a very definite reason. Under the plenum system the air carries with it the heat, no other means of warming the building being provided. The heat so carried having then to replace all that which is lost through the walls and windows, the air in the conduits must always be hotter than it is required to be in the wards. If then the outlet were at the top a short circuit would quickly be established, and much of the air in the room would remain unchanged. We see then that so far as concerns the plenum system as carried out at present—that is, without local heating appliances in the form of radiators—bottom outlets are essential if the heat is ever to reach the floor; while in the case of open fireplaces the bottom extraction which it provides is a mere accident, good merely so far as it utilises an otherwise waste force. But if one puts on one side the plenum system and all such incidental ventilation as takes place when open fires are in use (in regard to both of which there is definite reason for the outlet being low), there can be no doubt that the proper course is for ventilating outlets to be placed at the top of the room. Not that we must accept the view that the expired air floats up from the mouth of the patient and, arriving at the ceiling, goes straight out by the patent ventilator. Things are not by any means so simple as that. The warmth of the fire, the cooling by the walls and windows, and the diffusion of the gaseous particles, lead to such a general mixture of the air that some at least of what has been breathed once is breathed again, as, of course, happens also in the plenum system. The nearest approach to a theoretically perfect system is that in which the natural tendency of the expired air to float upwards is least interfered with, that, namely, in which the air escapes by outlets at the top of the room. If then we put out of present consideration the plenum system as being only requisite in special circumstances, we cannot doubt that the most complete and satisfactory form of ventilation for hospitals and living-rooms is that which combines open fireplaces with the warming of the incoming air, its removal at the level of the ceiling, and the provision of arrangements for perflation from window to window whenever the weather will allow of it.

